# Toxic Effects of Endocrine Disruptor Exposure on Collagen-Induced Arthritis

**DOI:** 10.3390/biom12040564

**Published:** 2022-04-10

**Authors:** Ramona D’Amico, Enrico Gugliandolo, Marika Cordaro, Roberta Fusco, Tiziana Genovese, Alessio Filippo Peritore, Rosalia Crupi, Livia Interdonato, Davide Di Paola, Salvatore Cuzzocrea, Daniela Impellizzeri, Rosalba Siracusa, Rosanna Di Paola

**Affiliations:** 1 Department of Chemical, Biological, Pharmaceutical and Environmental Sciences, University of Messina, 98166 Messina, Italy; rdamico@unime.it (R.D.); tiziana.genovese@unime.it (T.G.); aperitore@unime.it (A.F.P.); interdonatol@unime.it (L.I.); davide.dipaola@unime.it (D.D.P.); rsiracusa@unime.it (R.S.); 2 Department of Veterinary Science, University of Messina, 98168 Messina, Italy; egugliandolo@unime.it (E.G.); rcrupi@unime.it (R.C.); dipaolar@unime.it (R.D.P.); 3 Department of Biomedical, Dental and Morphological and Functional Imaging, University of Messina, 98125 Messina, Italy; marika.cordaro@unime.it; 4 Department of Clinical and Experimental Medicine, University of Messina, 98125 Messina, Italy; rfusco@unime.it

**Keywords:** endocrine disruptors, arthritis, inflammation, oxidative stress

## Abstract

Endocrine disruptors (EDs) are chemical substances capable of affecting endocrine system functioning and interfering with organ morphogenesis and physiological functions. The development and regeneration of bone tissues have a complex hormonal regulation, and therefore, bone tissue cells can be considered potential targets for endocrine disruptors. In that regard, the aim of this research was to investigate the impact of ED exposure on the inflammatory response and oxidative stress in an experimental model of collagen-induced arthritis (CIA). Arthritis was induced by an emulsion of type II collagen (CII) and complete Freund’s adjuvant, which was administered intradermally on days 0 and 21. Mice from day 21 to day 35 received the following EDs by oral gavage: cypermethrin (CP), diethyl phthalate (DEP), vinclozolin (VCZ), 17α-ethinylestradiol (EE), perfluorooctanesulfonic acid (PFOS) and atrazine (ATR). ED exposure caused worsening of clinical signs (erythema and edema in the hind paws), histological and radiographic changes, as well as behavioral deficits, induced by CII injections. Furthermore, ED exposure significantly increased the degree of inflammation and oxidative damage induced by arthritis; this upregulation was more evident after exposure to ATR than to other EDs. The results from our study suggest that exposure to EDs may play a deleterious role in the progression of RA; therefore, exposure to EDs should be limited.

## 1. Introduction

Exposure to endocrine disruptors (EDs) has increased in recent years, and the impact of these chemicals on human health is increasingly concerning [1]. EDs are commonly found in the environment and are derived from industrial and agricultural sources, including pesticides, herbicides, and other chemicals used in the plastics industry and in consumer products. EDs have gained interest in human physiopathology, as they may interfere with the synthesis, secretion, transport, metabolism, receptor binding, or elimination of endogenous hormones, altering the endocrine and homeostatic systems [2,3]. Indeed, epigenetic changes, such as DNA methylation and/or acetylation and histone modifications, appear to be involved in mechanisms related to endocrine disruption [4,5,6]. The choice of focused categories herein presented is relative to the vast diversity of EDs. The principal categories are classified as (1) food contact materials such as bisphenol A, (2) chemicals in products such as diethyl phthalate (DEP) or perfluorooctanesulfonic acid (PFOS), (3) synthetic estrogen such as 17α-ethinylestradiol (EE), and (4) pesticides such as cypermethrin (CP), vinclozolin (VCZ), and atrazine (ATR) [7]. Among them, ATR in particular is the most commonly found pesticide in surface and groundwater, where it tends to persist, as it has a half-life of 95–350 days and is resistant to degradation [8]. The exact molecular processes underlying ED effect have not been fully understood, although they seem to alter hormone balance, resulting in endocrine and reproductive system disorders. As endocrine hormones are known to play important roles in regulating immune responses, EDs may influence immune responses by various mechanisms. In vitro and in vivo studies have indicated that EDs can influence the immune system by acting on many levels of the immune regulatory network, including cellular and humoral response, survival, maturation, and cytokine synthesis of immune cells [9,10,11]. Additionally, several endocrine hormones are involved or associated with inflammatory joint diseases such as rheumatoid arthritis (RA), suggesting their interference with bone metabolism [12,13]. The development and regeneration of bone tissues are regulated by a complex of hormones, growth factors, and prostaglandins having a direct or indirect influence on the mineral exchange and the processes of cell proliferation and differentiation [11,14]. Therefore, EDs can cause various alterations in the metabolism of bone tissues and in their structural and functional organization [15].

In light of the above, this study was designed to evaluate how exposure to several EDs could affect the development of RA, an autoimmune disease that triggers pain and inflammation, as well as articular cartilage degradation and bone erosion. As it shares many of the cellular and humoral immune characteristics of human RA [16], collagen-induced arthritis (CIA) in mice has shown to be a suitable experimental model of RA.

## 2. Materials and Methods

### 2.1. Animals

DBA/1 J mice (male; 27–30 gr Envigo, Italy) were used in these studies. Mice were housed in individual cages (5 per cage) and posted in a controlled environment. The Review Board for the care of animals of the University of Messina approved the study (ethical protocol code: n 904/2021-PR). All in vivo experiments followed the new directives of the USA, Europe, Italy, and the ARRIVE guidelines.

### 2.2. CIA-Induced RA and Treatments

Type II chicken collagen (CII) was mixed in 0.01 M acetic acid at a concentration of 2 mg/mL. Complete Freund’s adjuvant (CFA) was mixed with Mycobacterium tuberculosis H37Ra at a concentration of 2 mg/mL. Animals were anesthetized with intraperitoneal xylazine and ketamine (0.16 and 2.6 mg/kg body weight, respectively). Mice were subjected to two intradermally injections at the base of the tail with 100 μL of emulsion (containing an equal volume of CII and CFA) on day 0 and day 21 [17].

Mice received by oral gavage the following EDs: CP (15 mg/kg), DEP (2 μg/mL), VCZ (100 mg/kg), EE (1 μg/kg), PFOS (10 mg/kg), and ATR (25mg/kg). Mice were exposed to EDs every day, starting from day 21 to day 35. The dose used and the route of CII and EDs were based on previous in vivo studies in the literature [8,17,18,19,20,21,22,23,24,25,26].

### 2.3. Experimental Groups

Mice were randomly divided into the following groups (n = 12 for each group):

− CIA: rats were subjected to CIA as described above;
− CIA + CP: same as the CIA group, and CP (15 mg/kg) was administered;
− CIA + DEP: same as the CIA group, and DEP (2 μg/mL) was administered;
− CIA + VCZ: same as the CIA group, and VCZ (100 mg/kg) was administered;
− CIA + EE: same as the CIA group, and EE (1 μg/kg) was administered;
− CIA + PFOS: same as the CIA group, and PFOS (10 mg/kg) was administered;
− CIA + ATR: same as the CIA group, and ATR (25 mg/kg) was administered;
− Sham groups = mice received two injections of 100 uL of 0.01 M acetic acid instead of the emulsion. Then, animals were orally administered with either vehicle or CP, DEP, VLZ, EE, PFOS, or ATR every day, starting from day 21 to day 35.

On day 35, paws and knees were collected for histological and biochemical investigation.

### 2.4. Behavioral Tests

After the training sessions, all tests were conducted on day 0 before CIA induction and subsequently on days 21, 25, 30, and 35, which included the following tests:

− Rotarod test to assess locomotor abilities, as previously described [27];
− Hotplate testing to evaluate pain sensitivity, as previously described [28];
− Thermal hyperalgesia to determine hyperalgesic responses to heat, as previously described [17].

### 2.5. Clinical Severity of CIA

A macroscopic scoring system was used to measure the progression of arthritis in all groups [29]. The arthritic index for each animal was calculated by adding the four scores of individual paws. Additionally, plethysmometry (model 7140; Ugo Basile) was used to quantify the variation in the paw volume for determining clinical severity [30].

### 2.6. Radiographic Analysis

Radiography was performed by X-ray (Bruker FX Pro instrument, Milan, Italy), as previously described. The radiographic criteria used to evaluate the hindlimbs were as follows: score 0, no bone damage; score 1, tissue swelling and edema; score 2, joint erosion; score 3, bone erosion and osteophyte formation [17].

### 2.7. Haematoxylin/Eosin (H/E) and Toluidine Blue Staining

Knee tissue sections (7 μm) were stained H/E and toluidine blue studied using a Leica DM6 microscope associated with Leica LAS X Navigator software (Leica Microsystems SpA, Milan, Italy). For the histological score, the morphological criteria were considered using a scale from 0 to 5 (0, no changes; 1, minimal changes; 2, mild changes; 3, moderate changes; 4, marked changes; 5, severe changes) for following parameters: inflammation, pannus, and bone resorption [17,31,32]. For cartilage damage score, the following morphological criteria were considered: 0, no changes; 1, minimal changes; 2, mild changes; 3, moderate changes; 4, marked changes; 5, severe changes [32].

### 2.8. Staining of Mast Cells

Toluidine blue stain was performed in the paw section as described previously [27] to identify mast cells. Sections were observed at a magnification of 40×, using a Leica DM6 microscope associated with Leica LAS X Navigator software (Leica Microsystems SpA, Milan, Italy).

### 2.9. Enzyme-Linked Immunosorbent Assay (ELISA)

Blood was withdrawn and centrifuged at 3000 rpm (4 °C, 10 min, twice). Levels of tumor necrosis factor-α (TNF-α), interleukin (IL)-6, IL-1β, IL-17A, and prostaglandin E2 (PGE2) were evaluated in the plasma from all groups, as previously described [17,33,34,35]. The assay was carried out by using a colorimetric commercial ELISA kit (Calbiochem-Novabiochem Corporation, Milan, Italy).

Levels of chemokines MIP-1α and MIP-2 were also measured in the aqueous joint extracts, as previously described [36]. In brief, joint tissues were prepared by first removing the skin and separating the limb below the ankle joint. Joint tissues were homogenized on ice in 3 mL of lysis buffer (PBS containing 2 mM phenylmethylsulfonyl fluoride and 0.1 mg/mL (final concentration) each of aprotinin, antipain, leupeptin, and pepstatin A) using a Polytron homogenizer (Brinkmann Instruments, Westbury, NY, USA). The homogenized tissues were then centrifuged at 2000× *g* for 10 min. Supernatants were sterilized with a Millipore filter (0.2 μm) and stored at −80 °C until analyzed. The extracts usually contained 0.2 to 1.5 mg of protein/mL, as measured by a protein assay kit (Thermo Fisher Scientific, Waltham, MA, USA). The levels of MIP-1α and MIP-2 were quantified using a modification of a double ligand method, as described previously [37].

The expressions of superoxide dismutase (SOD) and glutathione peroxidase (GSH-Px) system were measured, as previously described [36]. Briefly, the hind paw tissues were homogenized with 100 μL ice-cold tissue RIPA lysis buffer. The homogenate was incubated at 4 °C for 30 min and centrifuged at 12,000× *g* at 4 °C for 20 min. The supernatant was obtained as the total protein extract. SOD and GSH-Px expressions were measured using ELISA kits (R&D Systems, Minneapolis, MN, USA) following the manufacturer’s instructions [38]. The amount of hydrogen peroxide (H_2_O_2_) was estimated using the Hydrogen Peroxide Assay Kit (Abcam, Cambridge, MA, USA) according to the manufacturer’s instructions [38].

### 2.10. Myeloperoxidase (MPO) Assay and Thiobarbituric Acid-Reactant Substances Measurement (MDA Levels)

Neutrophil infiltration to the inflamed joints was indirectly quantitated using an MPO assay [39], as previously indicated [17]. Each piece of tissue was homogenized in a solution containing 0.5% hexadecyltrimethylammonium bromide (HTAB) dissolved in 10 mM potassium phosphate buffer (pH 7) and centrifuged for 30 min at 20,000× *g* at 4 °C. An aliquot of the supernatant was then allowed to react with a solution of tetra-methyl-benzidine (1.6 mM) and 0.1 mM H_2_O_2_. The rate of change in absorbance was measured spectrophotometrically at 650 nm. MPO activity was defined as the quantity of enzyme degrading 1 μmol of peroxide min at 37 °C and was expressed in units per gram weight of wet tissue.

Malondialdehyde (MDA) is considered a good marker of lipid peroxidation and was determined in paw tissue, as previously described [33,40,41]. Briefly, paw tissue was homogenized in a 1.15% KCl solution. An aliquot (100 μL) of the homogenate was added to a reaction mixture containing 200 μL of 8.1% SDS, 1500 μL of 20% acetic acid (pH 3.5), 1500 μL of 0.8% thiobarbituric acid, and 700 μL of distilled water. Samples were then boiled for 1 h at 95 °C and centrifuged at 3000× *g* for 10 min. The absorbance of the supernatant was measured by spectrophotometry at 650 nm.

### 2.11. Immunohistochemical Analysis of Cyclooxygenase (COX)-2

Immunohistochemical analysis was performed as previously described [42]. Sections were incubated with the following primary antibodies: anti-COX-2 (Santa Cruz Biotechnology (SCB), Milan, Italy, sc-32258). Images were collected using a Leica DM6 microscope (Leica Microsystems SpA, Milan, Italy) following a typical procedure. The histogram profile is related to the positive pixel intensity value obtained.

### 2.12. Materials

Cypermethrin (Lot# BCCB1117), diethyl phthalate (Lot #BCBZ8989), vinclozolin (Lot #BCBZ5052), 17α-ethinylestradiol (Lot #WXBC9894V), perfluorooctanesulfonic acid (#0000091932), atrazine (Lot# BCBZ3835) were obtained from Sigma-Aldrich INC. P.O. (St. Louis, MO, 63178, USA). Unless otherwise stated, all compounds were purchased from Sigma-Aldrich. All solutions used for in vivo infusions were prepared using nonpyrogenic saline (0.9% NaCl; Baxter Healthcare Ltd., Thetford, Norfolk, UK).

### 2.13. Statistical Evaluation

All values are expressed as mean ± standard deviation (SD) of N observations. The images shown are representative of the least 3 experiments performed on different experimental days on tissue sections collected from all animals. For in vivo studies, N represents the number of animals used. The results were analyzed by one-way ANOVA followed by a Bonferroni post hoc test for multiple comparisons. A *p*-value less than 0.05 was considered significant.

## 3. Results

### 3.1. Impact of ED Exposure on Behavioral Function

**Figure 1:**
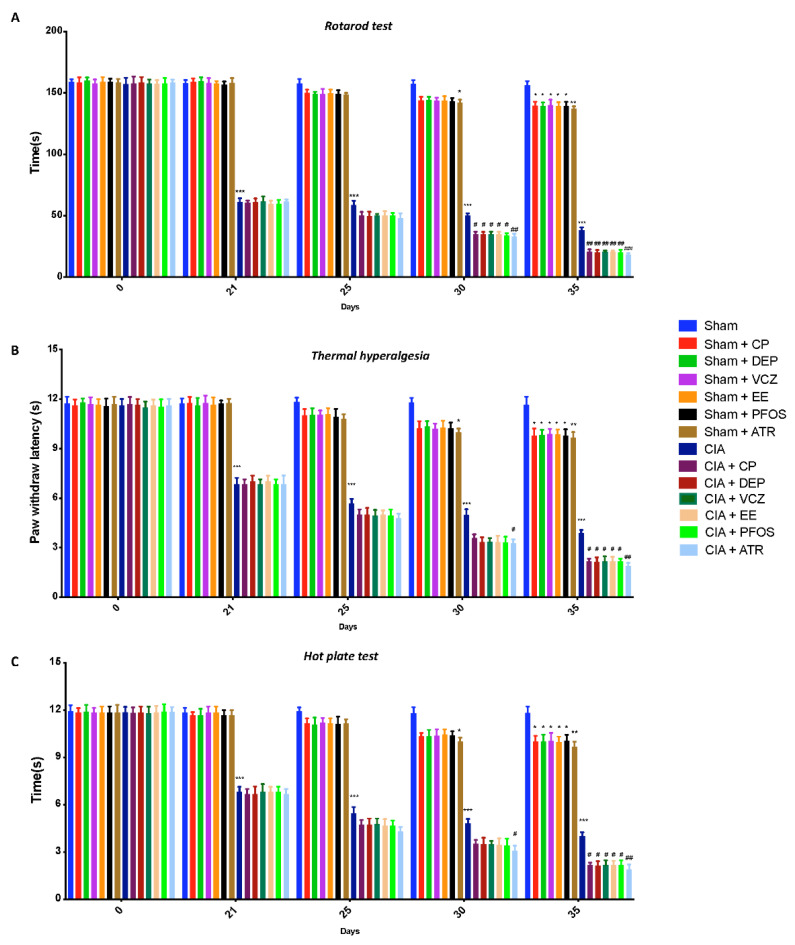
Behavioral analysis: rotarod test for locomotor ability (**A**); plantar test for thermal hyperalgesia (**B**); hot-plate test for pain sensitivity (**C**). * *p* < 0.05 vs. sham; ** *p* < 0.01 vs. sham; *** *p* < 0.001 vs. sham; ^#^ *p* < 0.05 vs. CIA; ^##^ *p* < 0.01 vs. CIA; ^###^ *p* < 0.001 vs. CIA.

First, we subjected mice from all groups to behavioral tests. No differences were observed in locomotor ability (Figure 1A), thermal hyperalgesia (Figure 1B), and pain sensitivity (Figure 1C) in all animals before CIA induction, while the groups subjected to only ED exposure showed minimal changes. Arthritis mice exhibited a significant motor dysfunction, as indicated by a decrease in time spent on rotarod in the CIA-alone group. Simultaneous exposure to both CII and EDs, especially to ATR, notably increased motor impairment (Figure 1A). Furthermore, animals became hypersensitive to heat stimuli (thermal hyperalgesia) around day 21 following the first CIA induction, as demonstrated by a substantial reduction in hind paw withdrawal latency, with a maximum hypersensitive response reported in CIA-control mice between days 30 and 35 post-immunization (Figure 1B). This CIA-induced hyperalgesia was markedly increased by daily exposure to EDs, in particular to ATR. Then, pain sensitivity was tested by subjecting mice to a hot-plate test (Figure 1C). On days 21–25, and even more during days 30–35 after CIA induction, vehicle animals displayed increased pain sensitivity, compared with all sham groups. Again, we found that ATR-treated mice were more susceptible to pain sensitivity than to other EDs, at day 35 post-immunization.

### 3.2. Impact of ED Exposure on Clinical Signs and Body Weight

**Figure 2:**
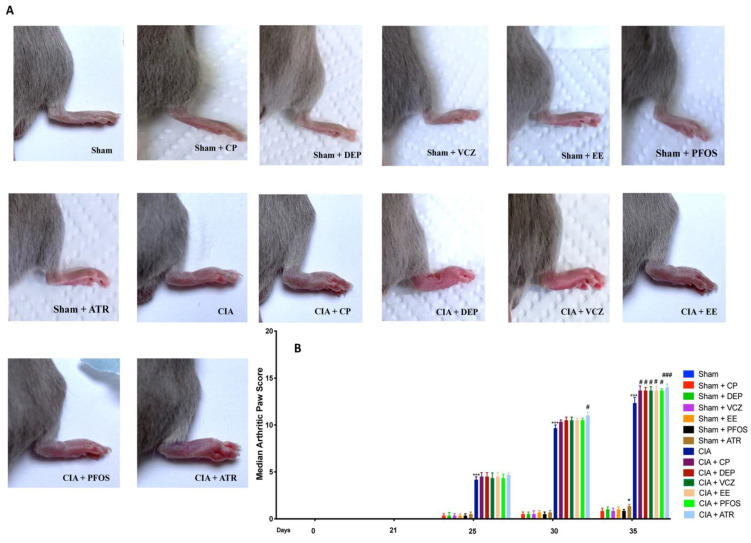
Macroscopical analysis of paw from all groups (**A**); median arthritic paw score at days 0, 21, 25, 30, 35 (**B**). * *p* < 0.05 vs. sham; *** *p* < 0.001 vs. sham; ^#^ *p* < 0.05 vs. CIA; ^###^ *p* < 0.001 vs. CIA.

Macroscopic signs of CIA, such as periarticular erythema and swelling, increased in frequency and severity in a time-dependent manner, with maximum arthritis indices observed between days 30 and 35 after simultaneous CII and ATR administration, more prominent than the other ED-treated mice. No clinical signs of CIA were observed in the paws of sham groups during the evaluation period (Figure 2A,B).

**Figure 3:**
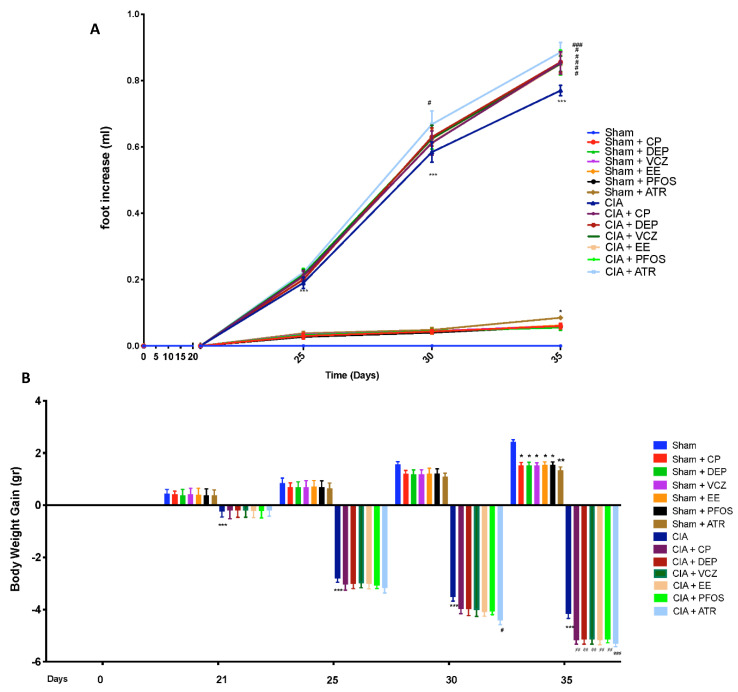
Variation in paw volume (**A**); gain in body weight (**B**). * *p* <0.05 vs. sham; ** *p* < 0.01 vs. sham; *** *p* < 0.001 vs. sham; ^#^ *p* < 0.05 vs. CIA; ^##^ *p* < 0.01 vs. CIA; ^###^ *p* < 0.001 vs. CIA.

In addition, Figure 3A demonstrated a time-dependent increase in paw volume (each value is the mean value of both hind paws). Exposure to EDs, in particular to ATR, enhanced foot increase, compared with the CIA-control group. No increases in hind-paw volume were observed in the sham group.

The gain in body weight was similar in sham and in CIA mice in the first week. From day 21, CIA mice gained less weight than sham groups, until day 35. Exposure to several Eds, and even more to ATR, caused a major decrease in body weight gain (Figure 3B).

### 3.3. Impact of ED Exposure on Radiographic Analysis

**Figure 4:**
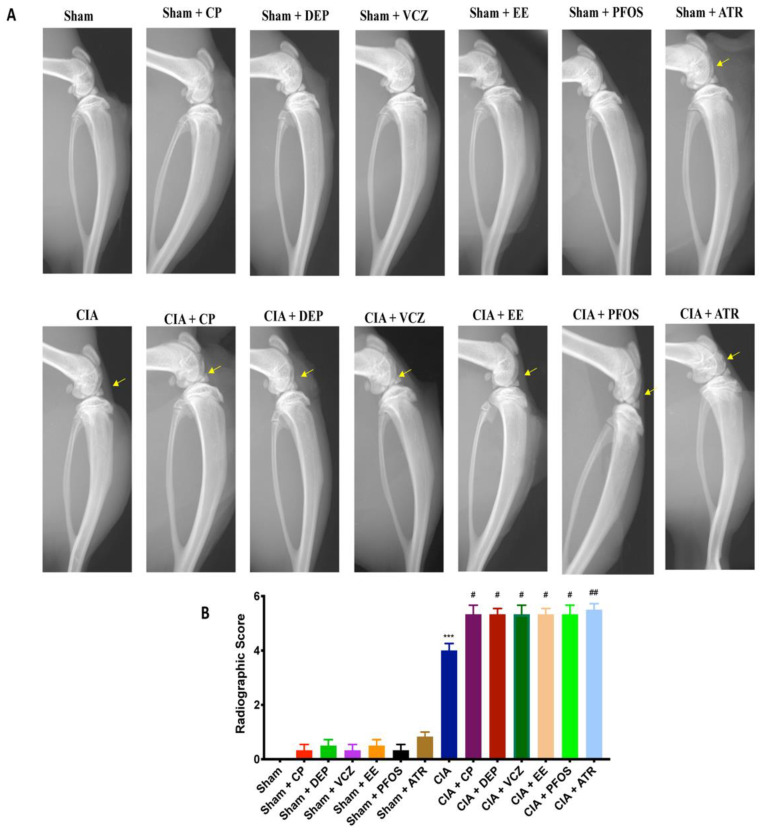
Radiography of knee joint from all groups (**A**); radiographic score (**B**). *** *p* < 0.001 vs. sham; ^#^ *p* < 0.05 vs. CIA; ^##^ *p* < 0.01 vs. CIA.

We performed a radiographic examination on day 35. Sham animals showed no evidence of bone resorption. Radiography of knee joint and femoral growth plate revealed bone erosion in the femur collected from CIA-control mice, and even more so after simultaneous exposure with EDs. ATR-treated mice showed more significantly increased joint damage, compared with the other groups (Figure 4A,B).

### 3.4. Impact of ED Exposure on Histopathological Analysis

**Figure 5:**
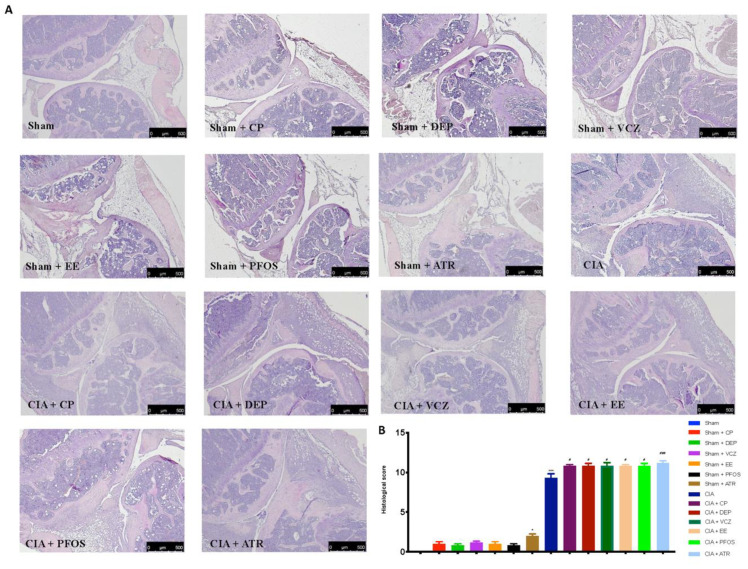
Histological evaluation of knee from all groups (**A**); histological score (**B**). A 2,5× magnification is shown (500-µm scale bar). * *p* < 0.05 vs. sham; *** *p* < 0.001 vs. sham; ^#^ *p* < 0.05 vs. CIA; ^###^ *p* < 0.001 vs. CIA.

H&E and toluidine blue staining were performed to evaluate the histopathological alteration induced by CII injection. The results of both stainings showed that single exposure to EDs could damage the joints in some way but not so severe as CII-adjuvant. In particular, the groups treated alone with DEP, EE, PFOS, and ATR showed alterations in the joint structure, compared with the sham group, as demonstrated by H&E staining. By contrast, mice treated simultaneously with EDs and CII revealed more obvious signs of arthritis (bone erosion of joint) than those groups treated with single molecules (Figure 5A,B).

**Figure 6:**
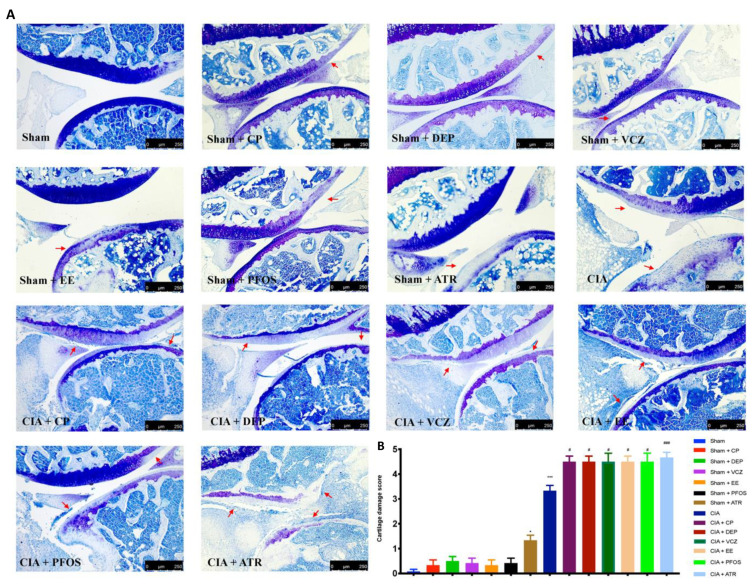
Toluidine blue staining for knee from all groups (**A**); cartilage damage score (**B**). A 10× magnification is shown (250 µm scale bar). Red arrows indicated cartilage damage. * *p* < 0.05 vs. sham; *** *p* < 0.001 vs. sham; ^#^ *p* < 0.05 vs. CIA; ^###^ *p* < 0.001 vs. CIA.

We also observed loss of proteoglycan and cartilage integrity by toluidine blue staining. Similar to the above result, the cartilages were also potentially affected by EDs alone such as CP, DEP, VCZ, PFOS, and ATR but not so severe as in the CIA group. In particular, after CII injections, ATR exposure showed more evident joint inflammation and cartilage degradation than other groups (Figure 6A,B).

### 3.5. Impact of ED Exposure on Mast Cell Degranulation

**Figure 7:**
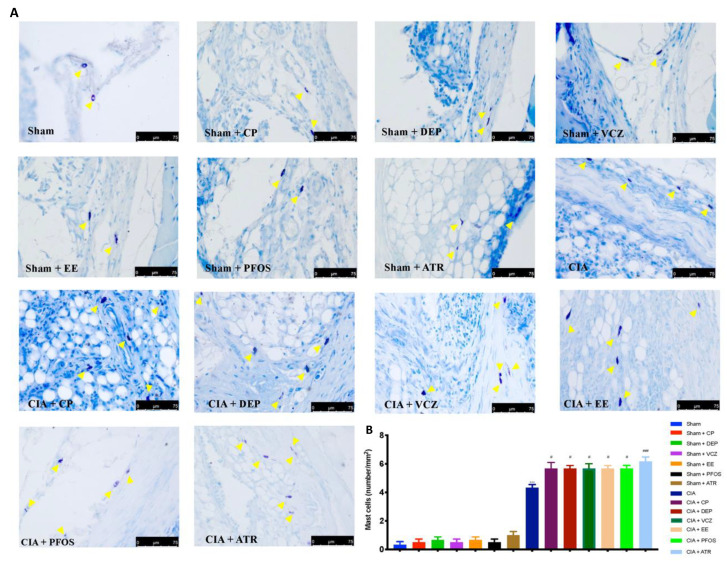
Evaluation of mast cells by toluidine blue staining (**A**); mast cell count (**B**). A 40× magnification is shown (75 µm scale bar). Yellow arrows indicated mast cells. *** *p* < 0.001 vs. sham; ^#^ *p*< 0.05 vs. CIA; ^###^ *p* < 0.001 vs. CIA.

Toluidine blue staining was also performed to evaluate mast cell infiltration during CIA. A high number of mast cells were detected in the articular space, in the CIA group. The exposure to several Eds, and even more to ATR, caused a major increase in the number of mast cells. No resident or few mast cells were found in the joint sections from all sham groups (Figure 7A,B).

### 3.6. Impact of ED Exposure on Cytokine and Chemokine Levels and Neutrophil Infiltration

**Figure 8:**
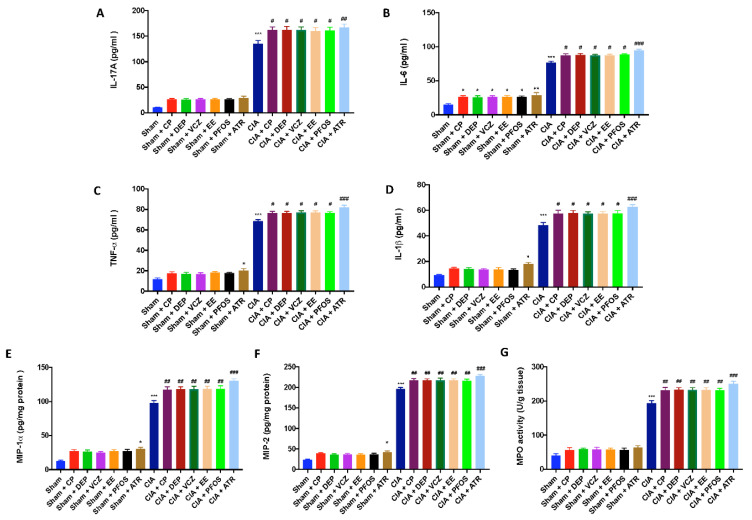
Levels of cytokines: IL-17A (**A**); IL-6 (**B**); IL-1β (**C**); TNF-α (**D**). Levels of chemokines: MIP-lα (**E**) and MIP-2 (**F**). MPO assay for neutrophil infiltration (**G**). * *p* < 0.05 vs. sham; ** *p* < 0.01 vs. sham; *** *p* < 0.001 vs. sham; ^#^ *p* < 0.05 vs. CIA; ^##^ *p* < 0.01 vs. CIA; ^###^ *p* < 0.001 vs. CIA.

To investigate the regulation of cytokine secretion 35 days after immunization, we analyzed the levels of IL-17-A (Figure 8A), IL-6 (Figure 8B), IL-1β (Figure 8C), and TNF-α (Figure 8D) in the plasma. A substantial increase in proinflammatory cytokines was found in CIA-control mice. Similar results were observed for chemokines MIP-lα (Figure 8E) and MIP-2 (Figure 8F) levels in the inflamed joint tissue. The increase in proinflammatory cytokine and chemokine production was more evident after simultaneous CII and ED administration, and to a more significant extent in the group exposed to ATR.

Neutrophil infiltration into the joint tissue was evaluated for MPO activity (Figure 8G). It was significantly elevated in CIA-control mice, compared with sham groups; while exposure to EDs, especially to ATR, increased MPO activity more significantly.

### 3.7. Impact of ED Exposure on Oxidative Stress and Lipid Peroxidation

**Figure 9:**
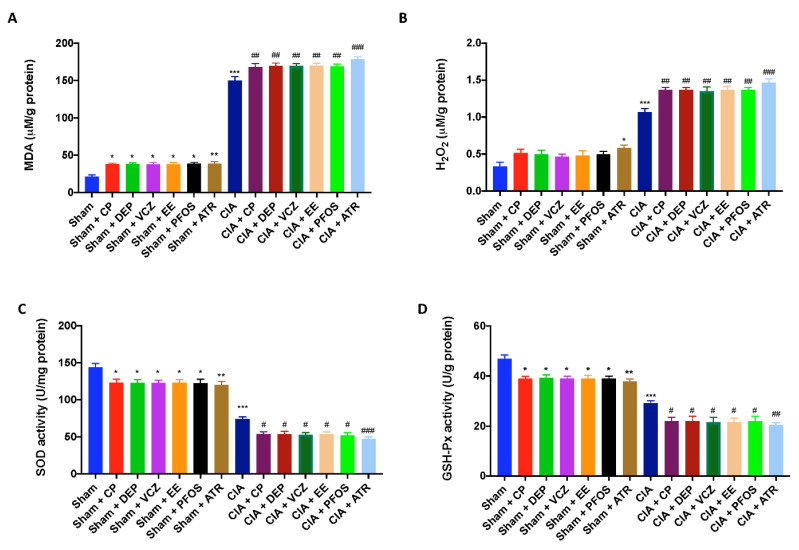
Levels of MDA (**A**); H_2_O_2_ (**B**); SOD (**C**); GSH-Px (**D**). * *p* < 0.05 vs. sham; ** *p* < 0.01 vs. sham; *** *p* < 0.001 vs. sham; ^#^ *p* < 0.05 vs. CIA; ^##^ *p* < 0.01 vs. CIA; ^###^ *p* < 0.001 vs. CIA.

It has been suggested that the release of free radicals and oxidizing agents contributes to disease severity in RA. Our results confirmed that MDA (Figure 9A) and H_2_O_2_ (Figure 9B) concentrations were drastically increased after double exposure to CII and EDs, compared with single molecules. Again, ATR-treated mice were more susceptible to oxidative stress and lipid peroxidation than other groups. In line with this finding, the activity of antioxidant enzymes, such as SOD (Figure 9C) and GSH-Px (Figure 9D) in paw tissues, significantly dropped after both exposure to CII and EDs, and to a more significant extent in the group exposed to ATR.

### 3.8. Impact of ED Exposure on COX-2 and PGE2 Expression

**Figure 10:**
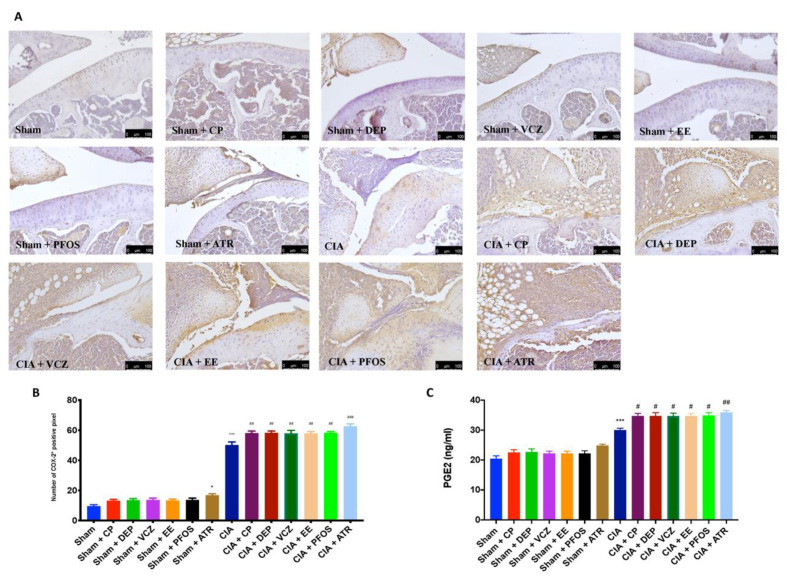
Immunohistochemical evaluation in knee joint for COX-2 expression (**A**); graphical quantification of COX-2 expression (**B**); levels of PGE2 in plasma (**C**). A 20X magnification is shown (100 µm scale bar). * *p* < 0.05 vs. sham; *** *p* < 0.001 vs. sham; ^#^ *p* < 0.05 vs. CIA; ^##^ *p* < 0.01 vs. CIA; ^###^ *p* < 0.001 vs. CIA.

Immunohistochemical analysis of the tibiotarsal joint sections revealed positive staining for COX-2 after ED exposure and CII administration alone, compared with sham mice. Instead, the exposure to EDs, particularly to ATR, notably intensified COX-2 expression after CII immunization (Figure 10A,B). Consequently, we revealed an important increase in PGE2 levels in arthritis mice, especially after ED exposure. Notably, we observed that exposure to ATR caused an even more significant upregulation of this prostaglandin (Figure 10C).

## 4. Discussion

EDs and their derivatives are considered non-natural chemical compounds, which affect functions of the endocrine system and consequently alter the regulatory systems that preserve cellular homeostasis [43]. EDs are frequently employed in the industry and agriculture, contributing significantly to environmental pollution due to their long decomposition periods, ranging from many hours to several days or months [15,44,45]. Among EDs, ATR stands out for its fairly long half-life (95–350 days) and chemical stability, representing the most frequently detected pesticide in surface and groundwater [46]. Thus, human beings face a new challenge due to the high risk of adverse health outcomes. Currently, the United States Environmental Protection Agency (EPA) and EU have restricted the use of chemicals suspected of causing harm to human health, as well as to farm animals and wildlife, groundwater, or the atmosphere [47,48]. The investigations conducted so far have established that prolonged exposure to EDs affects negatively the endocrine system, as well as growth and postnatal development, compromising with epigenetic mechanisms of morphogenetic events [34,49]. Furthermore, emerging research shows that EDs interfere with bone tissue formation and regeneration by altering the balance of osteoblast and osteoclast proliferation and differentiation [15]. However, at the same time, the contribution of EDs to the development of musculoskeletal and autoimmune pathologies is poorly studied. Therefore, our goal was to investigate the possible molecular mechanisms that underlie RA, an autoimmune disease characterized by chronic synovial joint inflammation and infiltration of activated immunoinflammatory cells, resulting in progressive cartilage degradation and bone erosion. Another hallmark of AR is the presence of proliferating cells with a tissue-infiltrating nature, forming hyperplastic tissue known as pannus [50]. To this end, we used an experimental model of CII-induced arthritis, and the animals were exposed to several EDs up to the 35th day, at the end of which behavioral tests were performed. CIA is associated with behavioral deficits, as demonstrated by an increase in allodynia, thermal hyperalgesia, and locomotor impairment by the rotarod test [17,50]. Besides increasing pain sensitivity and motor dysfunction, exposure to EDs, and even more to ATR, worsened macroscopic and radiographical alterations. Notably, the synergistic effect of CII and EDs is also reflected in histopathological alterations on the cartilage and bone, as highlighted by H/E and toluidine blue staining. Another feature of the arthritis joints is the activation of mast cells, which are an important source of cytokines and other inflammatory factors [51]. Simultaneous CII and ED administration induced an increase in both mast cell number and neutrophil infiltration into the inflamed joint tissue. Notably, we found that this increase was more marked in ATR-treated mice than in other groups. Analysis of the underlying molecular mechanisms of loss of bone integrity found that ED exposure led to overproduction of the proinflammatory cytokines in the arthritic joints. Specifically, the IL-17 cytokine family, particularly IL-17A, has been linked to the pathophysiology of RA in humans [52]. This cytokine triggers local inflammation and, in fact, induces synovial macrophages to generate proinflammatory cytokines, including IL-6 and IL-1β [53]. The latter, in turn, induces osteoclastogenesis by acting directly on osteoclast precursor cells, responsible for bone erosion [52,54]. Additionally, TNF-α has been found to mediate a series of immune effector functions that induce inflammatory responses and pannus formation, supporting the cartilage destruction and bone erosion of RA [55]. As expected, our results confirmed the upregulation of proinflammatory cytokines in ED groups and even higher in ATR-treated mice. Similar results were observed in chemokine production, such as MIP-1 and MIP-2, which are factors chemotactic for lymphocyte subset, suggesting a key role in the progression of joint inflammation, especially after ED exposure. Oxidative stress, described as an imbalance between ROS synthesis and antioxidants, contributes to inflammation and destruction of arthritic animal joints and RA patients [38,56,57]. In this regard, we found high levels of H_2_O_2_ and lipid peroxidation were present in arthritic animals, especially after exposure to EDs and even more to ATR. Additionally, our results also demonstrated a decrease in levels of antioxidant enzymes, such as SOD and GSH-Px in all groups following ED exposure. On the other hand, oxidative stress has emerged as a mechanism of ED toxicity. The release of ROS by synoviocytes, chondrocytes, and osteoblasts results in complex biochemical interactions with other biological molecules to promote pain and joint degeneration [58]. CII-induced AR was also found to lead to a significant rise in the expression of COX-2, an enzyme that converts arachidonic acid to PGE2, itself a mediator of inflammation and reactions leading to tissue injury [55,59]. In line with the literature, we found that COX-2 expression in infiltrating inflammatory mononuclear cells and subsynovial fibroblast-like cells was upregulated in the arthritis mice. Exposure to EDs, especially to ATR, showed a more marked increase in both COX-2 and PGE2 expressions, confirming their role in the progression of inflammation, joint pain, and cartilage damage. In summary, our data demonstrated that ED exposure causes increased destruction of cartilage and bone erosion during CIA. Notably, we observed that exposure to ATR worsened the CII-induced damage. However, the role of EDs in the involvement of other molecular pathways in CIA needs to be investigated. Another limitation of this study is the hormonal aspect, as the relationship between endocrine hormones and the spectrum of rheumatic conditions has long been discussed in the literature [58,59,60]. Sexual hormones (estrogens, androgens, prolactin) or hormones acting on the bone tissue (parathyroid hormone, vitamin D) are indeed involved in the pathogenesis of the main inflammatory arthritis, owing to their effects on the immune system, both stimulatory and inhibitory. A deeper investigation of the relationship between bone turnover, hormones, and immune and inflammatory responses will be the starting point for future studies.

## 5. Conclusions

The cellular mechanisms that underlie ED-exposure-induced toxicity are probably due to exacerbation of inflammation and oxidative stress, thus inducing an imbalance in cellular redox homeostasis and an overproduction of cytokines and chemokines. However, further studies are required to investigate the mechanisms by which EDs regulate other signaling pathways in CIA. The clinical relevance of real-life exposure to EDs still needs to be addressed, as identifying the risk associated with a single ED is complex. Humans are exposed to low doses of hundreds of chemicals, and adverse effects can develop latently, manifest later in life, or not appear at all in some people. Therefore, all precautions should be used to limit the impact of human exposure to EDs, especially in utero and postnatal development. Additional studies are required to understand mechanisms of toxicity and determine potential health risks associated with ED exposure.

## Data Availability

As a rule of our laboratory, the datasets used in the current study are available from the corresponding author (dimpellizzeri@unime.it) on reasonable request.

## References

[1] Yilmaz B., Terekeci H., Sandal S., Kelestimur F. (2020). Endocrine disrupting chemicals: Exposure, effects on human health, mechanism of action, models for testing and strategies for prevention. Rev. Endocr. Metab. Disord..

[2] Lauretta R., Sansone A., Sansone M., Romanelli F., Appetecchia M. (2019). Endocrine Disrupting Chemicals: Effects on Endocrine Glands. Front. Endocrinol..

[3] De Coster S., van Larebeke N. (2012). Endocrine-disrupting chemicals: Associated disorders and mechanisms of action. J. Environ. Public Health.

[4] Gore A.C., Chappell V.A., Fenton S.E., Flaws J.A., Nadal A., Prins G.S., Toppari J., Zoeller R.T. (2015). EDC-2: The Endocrine Society’s Second Scientific Statement on Endocrine-Disrupting Chemicals. Endocr. Rev..

[5] Koch C.A., Diamanti-Kandarakis E. (2015). Introduction to Endocrine Disrupting Chemicals—Is it time to act?. Rev. Endocr. Metab. Disord..

[6] D’Amico R., Monaco F., Fusco R., Siracusa R., Impellizzeri D., Peritore A.F., Crupi R., Gugliandolo E., Cuzzocrea S., Di Paola R. (2021). Atrazine Inhalation Worsen Pulmonary Fibrosis Regulating the Nuclear Factor-Erythroid 2-Related Factor (Nrf2) Pathways Inducing Brain Comorbidities. Cell Physiol. Biochem..

[7] Kuo C.H., Yang S.N., Kuo P.L., Hung C.H. (2012). Immunomodulatory effects of environmental endocrine disrupting chemicals. Kaohsiung J. Med. Sci..

[8] Popescu M., Feldman T.B., Chitnis T. (2021). Interplay Between Endocrine Disruptors and Immunity: Implications for Diseases of Autoreactive Etiology. Front. Pharmacol..

[9] Chalubinski M., Kowalski M.L. (2006). Endocrine disrupters—Potential modulators of the immune system and allergic response. Allergy.

[10] Tang M.W., Garcia S., Gerlag D.M., Tak P.P., Reedquist K.A. (2017). Insight into the Endocrine System and the Immune System: A Review of the Inflammatory Role of Prolactin in Rheumatoid Arthritis and Psoriatic Arthritis. Front. Immunol..

[11] Kelley K.W., Weigent D.A., Kooijman R. (2007). Protein hormones and immunity. Brain Behav. Immun..

[12] Al-Suhaimi E.A., Al-Jafary M.A. (2020). Endocrine roles of vitamin K-dependent- osteocalcin in the relation between bone metabolism and metabolic disorders. Rev. Endocr. Metab. Disord..

[13] Yaglova N.V., Yaglov V.V. (2021). Endocrine Disruptors as a New Etiologic Factor of Bone Tissue Diseases (Review). Sovrem Tekhnol. Med..

[14] Holmdahl R., Andersson M., Goldschmidt T.J., Gustafsson K., Jansson L., Mo J.A. (1990). Type II collagen autoimmunity in animals and provocations leading to arthritis. Immunol. Rev..

[15] Impellizzeri D., Siracusa R., Cordaro M., Peritore A.F., Gugliandolo E., D’Amico R., Fusco R., Crupi R., Rizzarelli E., Cuzzocrea S. (2020). Protective effect of a new hyaluronic acid -carnosine conjugate on the modulation of the inflammatory response in mice subjected to collagen-induced arthritis. Biomed. Pharmacother..

[16] Yang B., Zou W., Hu Z., Liu F., Zhou L., Yang S., Kuang H., Wu L., Wei J., Wang J. (2014). Involvement of oxidative stress and inflammation in liver injury caused by perfluorooctanoic acid exposure in mice. Biomed. Res. Int..

[17] Coban A., Filipov N.M. (2007). Dopaminergic toxicity associated with oral exposure to the herbicide atrazine in juvenile male C57BL/6 mice. J. Neurochem..

[18] Guerrero-Bosagna C., Covert T.R., Haque M.M., Settles M., Nilsson E.E., Anway M.D., Skinner M.K. (2012). Epigenetic transgenerational inheritance of vinclozolin induced mouse adult onset disease and associated sperm epigenome biomarkers. Reprod. Toxicol..

[19] European Food Safety Authority (2018). Statement on the impact of the harmonised classification on the conclusion on the peer review of the pesticide risk assessment of the active substance flutianil. EFSA J..

[20] Singh D., Irani D., Bhagat S., Vanage G. (2020). Cypermethrin exposure during perinatal period affects fetal development and impairs reproductive functions of F1 female rats. Sci. Total Environ..

[21] Saravanabhavan G., Walker M., Guay M., Aylward L. (2014). Urinary excretion and daily intake rates of diethyl phthalate in the general Canadian population. Sci. Total Environ..

[22] Leitz J., Kuballa T., Rehm J., Lachenmeier D.W. (2009). Chemical analysis and risk assessment of diethyl phthalate in alcoholic beverages with special regard to unrecorded alcohol. PLoS ONE.

[23] Cleuren A.C., Van der Linden I.K., De Visser Y.P., Wagenaar G.T., Reitsma P.H., Van Vlijmen B.J. (2010). 17alpha-Ethinylestradiol rapidly alters transcript levels of murine coagulation genes via estrogen receptor alpha. J. Thromb. Haemost..

[24] LaPlante C.D., Vandenberg L.N. (2017). Data describing lack of effects of 17alpha-ethinyl estradiol on mammary gland morphology in female mice exposed during pregnancy and lactation. Data Brief.

[25] Siracusa R., Fusco R., Cordaro M., Peritore A.F., D’Amico R., Gugliandolo E., Crupi R., Genovese T., Evangelista M., Di Paola R. (2020). The Protective Effects of Pre- and Post-Administration of Micronized Palmitoylethanolamide Formulation on Postoperative Pain in Rats. Int. J. Mol. Sci..

[26] Peritore A.F., Siracusa R., Fusco R., Gugliandolo E., D’Amico R., Cordaro M., Crupi R., Genovese T., Impellizzeri D., Cuzzocrea S. (2020). Ultramicronized Palmitoylethanolamide and Paracetamol, a New Association to Relieve Hyperalgesia and Pain in a Sciatic Nerve Injury Model in Rat. Int. J. Mol. Sci..

[27] Szabo C., Virag L., Cuzzocrea S., Scott G.S., Hake P., O’Connor M.P., Zingarelli B., Salzman A., Kun E. (1998). Protection against peroxynitrite-induced fibroblast injury and arthritis development by inhibition of poly(ADP-ribose) synthase. Proc. Natl. Acad. Sci. USA.

[28] Gugliandolo E., Peritore A.F., Impellizzeri D., Cordaro M., Siracusa R., Fusco R., D’Amico R., Paola R.D., Schievano C., Cuzzocrea S. (2020). Dietary Supplementation with Palmitoyl-Glucosamine Co-Micronized with Curcumin Relieves Osteoarthritis Pain and Benefits Joint Mobility. Animals.

[29] Mausset-Bonnefont A.L., Cren M., Vicente R., Quentin J., Jorgensen C., Apparailly F., Louis-Plence P. (2019). Arthritis sensory and motor scale: Predicting functional deficits from the clinical score in collagen-induced arthritis. Arthritis Res. Ther..

[30] Chen D.Y., Lin C.C., Chen Y.M., Chao Y.H., Yang D.H. (2017). Dextromethorphan Exhibits Anti-inflammatory and Immunomodulatory Effects in a Murine Model of Collagen-Induced Arthritis and in Human Rheumatoid Arthritis. Sci. Rep..

[31] Alavala S., Nalban N., Sangaraju R., Kuncha M., Jerald M.K., Kilari E.K., Sistla R. (2020). Anti-inflammatory effect of stevioside abates Freund’s complete adjuvant (FCA)-induced adjuvant arthritis in rats. Inflammopharmacology.

[32] Zhang Z., Cao C., Sun S., Xu Q. (2015). Selective spleen tyrosine kinase inhibition delays autoimmune arthritis in mice. Mol. Med. Rep..

[33] Min S.Y., Yan M., Kim S.B., Ravikumar S., Kwon S.R., Vanarsa K., Kim H.Y., Davis L.S., Mohan C. (2015). Green Tea Epigallocatechin-3-Gallate Suppresses Autoimmune Arthritis Through Indoleamine-2,3-Dioxygenase Expressing Dendritic Cells and the Nuclear Factor, Erythroid 2-Like 2 Antioxidant Pathway. J. Inflamm..

[34] Impellizzeri D., Esposito E., Di Paola R., Ahmad A., Campolo M., Peli A., Morittu V.M., Britti D., Cuzzocrea S. (2016). Erratum to: Palmitoylethanolamide and luteolin ameliorate development of arthritis caused by injection of collagen type II in mice. Arthritis Res. Ther..

[35] Impellizzeri D., Esposito E., Mazzon E., Paterniti I., Di Paola R., Morittu V.M., Procopio A., Britti D., Cuzzocrea S. (2011). Oleuropein aglycone, an olive oil compound, ameliorates development of arthritis caused by injection of collagen type II in mice. J. Pharmacol. Exp. Ther..

[36] Tang K.T., Lin C.C., Lin S.C., Wang J.H., Tsai S.W. (2021). Kurarinone Attenuates Collagen-Induced Arthritis in Mice by Inhibiting Th1/Th17 Cell Responses and Oxidative Stress. Int. J. Mol. Sci..

[37] Fusco R., Cordaro M., Genovese T., Impellizzeri D., Siracusa R., Gugliandolo E., Peritore A.F., D’Amico R., Crupi R., Cuzzocrea S. (2020). Adelmidrol: A New Promising Antioxidant and Anti-Inflammatory Therapeutic Tool in Pulmonary Fibrosis. Antioxidants.

[38] Cordaro M., Siracusa R., Fusco R., D’Amico R., Peritore A.F., Gugliandolo E., Genovese T., Scuto M., Crupi R., Mandalari G. (2020). Cashew (*Anacardium occidentale* L.) Nuts Counteract Oxidative Stress and Inflammation in an Acute Experimental Model of Carrageenan-Induced Paw Edema. Antioxidants.

[39] Ohkawa H., Ohishi N., Yagi K. (1979). Assay for lipid peroxides in animal tissues by thiobarbituric acid reaction. Anal. Biochem..

[40] D’Amico R., Fusco R., Cordaro M., Siracusa R., Peritore A.F., Gugliandolo E., Crupi R., Scuto M., Cuzzocrea S., Di Paola R. (2020). Modulation of NLRP3 Inflammasome through Formyl Peptide Receptor 1 (Fpr-1) Pathway as a New Therapeutic Target in Bronchiolitis Obliterans Syndrome. Int. J. Mol. Sci..

[41] Zoeller R.T., Brown T.R., Doan L.L., Gore A.C., Skakkebaek N.E., Soto A.M., Woodruff T.J., Vom Saal F.S. (2012). Endocrine-disrupting chemicals and public health protection: A statement of principles from The Endocrine Society. Endocrinology.

[42] Ying G.G., Toze S., Hanna J., Yu X.Y., Dillon P.J., Kookana R.S. (2008). Decay of endocrine-disrupting chemicals in aerobic and anoxic groundwater. Water Res..

[43] Kumar M., Sarma D.K., Shubham S., Kumawat M., Verma V., Prakash A., Tiwari R. (2020). Environmental Endocrine-Disrupting Chemical Exposure: Role in Non-Communicable Diseases. Front. Public Health.

[44] Genovese T., Siracusa R., Fusco R., D’Amico R., Impellizzeri D., Peritore A.F., Crupi R., Gugliandolo E., Morabito R., Cuzzocrea S. (2021). Atrazine Inhalation Causes Neuroinflammation, Apoptosis and Accelerating Brain Aging. Int. J. Mol. Sci..

[45] Kassotis C.D., Vandenberg L.N., Demeneix B.A., Porta M., Slama R., Trasande L. (2020). Endocrine-disrupting chemicals: Economic, regulatory, and policy implications. Lancet Diabetes Endocrinol..

[46] Kalofiri P., Balias G., Tekos F. (2021). The EU endocrine disruptors’ regulation and the glyphosate controversy. Toxicol. Rep..

[47] Alavian-Ghavanini A., Ruegg J. (2018). Understanding Epigenetic Effects of Endocrine Disrupting Chemicals: From Mechanisms to Novel Test Methods. Basic Clin. Pharmacol. Toxicol..

[48] Lite C., Raja G.L., Juliet M., Sridhar V.V., Subhashree K.D., Kumar P., Chakraborty P., Arockiaraj J. (2022). In utero exposure to endocrine-disrupting chemicals, maternal factors and alterations in the epigenetic landscape underlying later-life health effects. Environ. Toxicol. Pharmacol..

[49] Fusco R., Siracusa R., Peritore A.F., Gugliandolo E., Genovese T., D’Amico R., Cordaro M., Crupi R., Mandalari G., Impellizzeri D. (2020). The Role of Cashew (*Anacardium occidentale* L.) Nuts on an Experimental Model of Painful Degenerative Joint Disease. Antioxidants.

[50] Yoshimura S., Asano K., Nakane A. (2014). Attenuation of collagen-induced arthritis in mice by salmon proteoglycan. Biomed. Res. Int..

[51] McInnes I.B., Schett G. (2007). Cytokines in the pathogenesis of rheumatoid arthritis. Nat. Rev. Immunol.

[52] Alunno A., Carubbi F., Giacomelli R., Gerli R. (2017). Cytokines in the pathogenesis of rheumatoid arthritis: New players and therapeutic targets. BMC Rheumatol..

[53] Zhao Y., Liu F., Liu Y., Zhou D., Dai Q., Liu S. (2015). Anti-Arthritic Effect of Chebulanin on Collagen-Induced Arthritis in Mice. PLoS ONE.

[54] Esalatmanesh K., Jamali A., Esalatmanesh R., Soleimani Z., Khabbazi A., Malek Mahdavi A. (2022). Effects of N-acetylcysteine supplementation on disease activity, oxidative stress, and inflammatory and metabolic parameters in rheumatoid arthritis patients: A randomized double-blind placebo-controlled trial. Amino Acids.

[55] Liu J., Liu Y., Pan W., Li Y. (2021). Angiotensin-(1-7) attenuates collagen-induced arthritis via inhibiting oxidative stress in rats. Amino Acids.

[56] Salaffi F., Giacobazzi G., Di Carlo M. (2018). Chronic Pain in Inflammatory Arthritis: Mechanisms, Metrology, and Emerging Targets-A Focus on the JAK-STAT Pathway. Pain Res. Manag..

[57] Kunisch E., Jansen A., Kojima F., Loffler I., Kapoor M., Kawai S., Rubio I., Crofford L.J., Kinne R.W. (2009). Prostaglandin E2 differentially modulates proinflammatory/prodestructive effects of TNF-alpha on synovial fibroblasts via specific E prostanoid receptors/cAMP. J. Immunol..

[58] Villares R., Criado G., Juarranz Y., Lopez-Santalla M., Garcia-Cuesta E.M., Rodriguez-Frade J.M., Leceta J., Lucas P., Pablos J.L., Martinez A.C. (2018). Inhibitory Role of Growth Hormone in the Induction and Progression Phases of Collagen-Induced Arthritis. Front. Immunol..

[59] Cutolo M., Seriolo B., Villaggio B., Pizzorni C., Craviotto C., Sulli A. (2002). Androgens and estrogens modulate the immune and inflammatory responses in rheumatoid arthritis. Ann. N. Y. Acad. Sci..

[60] Bertoldo E., Adami G., Rossini M., Giollo A., Orsolini G., Viapiana O., Gatti D., Fassio A. (2021). The Emerging Roles of Endocrine Hormones in Different Arthritic Disorders. Front. Endocrinol..

